# Sub-acute Cardiac Tamponade as an Early Clinical Presentation of Childhood Systemic Lupus Erythematosus: A Case Report

**DOI:** 10.7759/cureus.3478

**Published:** 2018-10-22

**Authors:** Anum Umer, Shoaib Bhatti, Shafaq Jawed

**Affiliations:** 1 Internal Medicine, The Indus Hospital, Karachi, PAK; 2 Pediatrics, National Institute of Child Health, Karachi, PAK; 3 Surgery, Jinnah Sindh Medical University, Karachi, PAK

**Keywords:** cardiac tamponade, systemic lupus erythematosus (sle), childhood sle, autoimmunity

## Abstract

Systemic lupus erythematosus (SLE) is a chronic autoimmune disease affecting multiple systems by the process of inflammation and formation of auto-antibodies. When it presents in childhood, it is referred to as childhood systemic lupus erythematosus (cSLE). Cardiac tamponade is a rare but potentially lethal complication of cSLE, even rarer as an initial presentation. Sub-acute cardiac tamponade (medical tamponade) is a non-emergent type of cardiac tamponade which develops slowly over time and does not necessarily present with acute distress.

We present the case of an 11-year-old girl who presented to the emergency department with complaints of intermittent fever, periorbital puffiness, abdominal distension, and swelling on the hands and feet. She was not in any acute distress but was vitally unstable. Cardiovascular examination revealed muffled heart sounds. Chest examination further revealed decreased breathing sounds on the left side with dull notes on percussion. Abdominal examination revealed positive shifting dullness with a distended abdomen.

Blood investigations were ordered which revealed anemia and thrombocytopenia. Chest X-ray showed an enlarged cardiac silhouette. Urine detailed report showed proteinuria and hematuria. Further investigations revealed the autoimmune root of the disease. Echocardiography was ordered which showed a large collection of fluid around the posterior aspect of heart with the concomitant collapse of atrial chambers suggestive of cardiac tamponade. A diagnosis of sub-acute cardiac tamponade secondary to childhood SLE was made. The patient was started on pulse therapy of methylprednisolone followed by a low-dose regime of mycophenolate mofetil. The patient was also provided with positive pressure ventilation, hemodialysis, and invasive cardiovascular monitoring along with the instillation of intravenous fluid supplements. To our knowledge, cases of sub-acute cardiac tamponade as the only and early clinical manifestation in childhood SLE are very rare.

## Introduction

Clinical presentation of systemic lupus erythematosus (SLE) is quite diverse as it affects almost all organ systems of the human body. SLE occurs in both children and adults but it is most often seen in women of reproductive age. The most commonly affected organs are skin, joints, kidneys, blood vessels, and hematopoietic cells [1].

Childhood systemic lupus erythematosus (cSLE) constitutes approximately 20% of total SLE cases [2]. cSLE is usually diagnosed in adolescence and is rare before five years of age with the median age of diagnosis being 11-12 years [1]. The incidence of cSLE has been reported as 0.28-2.22 per 100,000 children and prevalence of 1-6 per 100,000 children, with higher frequencies in non-caucasian populations [1,3]. The heart is a commonly affected organ in all forms of SLE including cSLE. Pericarditis and pericardial effusion are the two well-recognized complications of cSLE, but cardiac tamponade is an unusual occurrence and even rarer as an initial presentation of SLE. Acute cardiac tamponade is a medical emergency but sub-acute cardiac tamponade is not always an emergency [4-5].

Here, we present a case of cSLE which involves an 11-year-old girl with sub-acute tamponade as an early clinical sign of cSLE. This, to our knowledge, has been very rarely reported and presents with different manifestations than in our case [6].

An important relationship to investigate is whether cardiac tamponade is a rare occurrence in cSLE or a rare occurrence only as an initial presentation. It is known that in adult SLE, cardiac tamponade is rare both as a complication and also as an initial presentation. However, it is still unknown if this association is also true in cases of cSLE.

## Case presentation

An 11-year-old female was admitted to the emergency department with complaints of intermittent fever for over one month, periorbital puffiness for four days, and abdominal distension with swelling on hands and feet for one day. In the hospital, she developed periorbital bruising and rashes on the extensor surfaces followed by the trunk over a period of four days. The patient experienced difficulty in walking due to a history of joint pain which was profound at the knee joint. However, there was no history of morning stiffness.

She denied photosensitivity, chest pain, shortness of breath, hair loss, oral ulcers, weight loss, fatigue, and any medication use. There was no known family history of autoimmune diseases. The patient reported having suffered from measles seven months back.

On examination, she was a tall, lean-built child, lying on bed, oriented to time, person, and place. Her vital signs on arrival were as follows: blood pressure, 142/110 mmHg (reference, 120/80 mmHg); pulse, 154 bpm (reference range, 75 to 118 bpm); and temperature, 37.5^o^C (reference range, 36.5 to 37.5^o^C). Her height was measured as 130 cm (at third centile) and weight, 25 kg below the third centile. The patient appeared sick-looking, with signs of pallor, conjunctival hemorrhage, and bruises around the eyes. Palpable purpura and petechiae over the extensor surfaces were seen whereas edema of the hands and feet was noted. Central nervous system (CNS) examination revealed 15/15 on the Glasgow Coma Scale (GCS). Cardiovascular examination revealed muffled heart sounds and good volume pulse. Chest examination revealed decreased breathing sounds on the left side of the chest with dull notes on percussion. Abdominal examination revealed positive shifting dullness with a distended abdomen.

Diagnostic criteria

Suspicion of SLE was raised on clinical findings, therefore, work-up for SLE was ordered. The updated diagnostic criteria of SLE was used which the patient fulfilled. Diagnosis of SLE is made if four out of 17 (including at least one of the eleven clinical and one of the six immunological) “Systemic Lupus International Collaborating Clinics (SLICC) classification criteria for systemic lupus erythematosus” are present. Biopsy-proven lupus nephritis with a positive antinuclear antibody (ANA) or anti-double-stranded deoxyribonucleic acid antibody (anti-dsDNA) also satisfies the diagnosis of SLE [1]. From the clinical criteria of SLICC, the following four were present in our patient: (i) serositis (muffled heart sounds and chest X-ray showing pericardial effusion); (ii) acute cutaneous lupus (conjunctival hemorrhage, bruises around eyes, palpable purpura, and petechiae over the extensor surfaces); (iii) renal (proteinuria, red blood cell casts); and (iv) raised spot urine protein/creatinine ratio.

From the six immunological criteria of SLICC, the following four were present: (i) ANA positive; (ii) C3 & C4 levels decreased; (iii) anti-dsDNA positive; and (iv) direct Coombs test positive.

**Figure 1:**
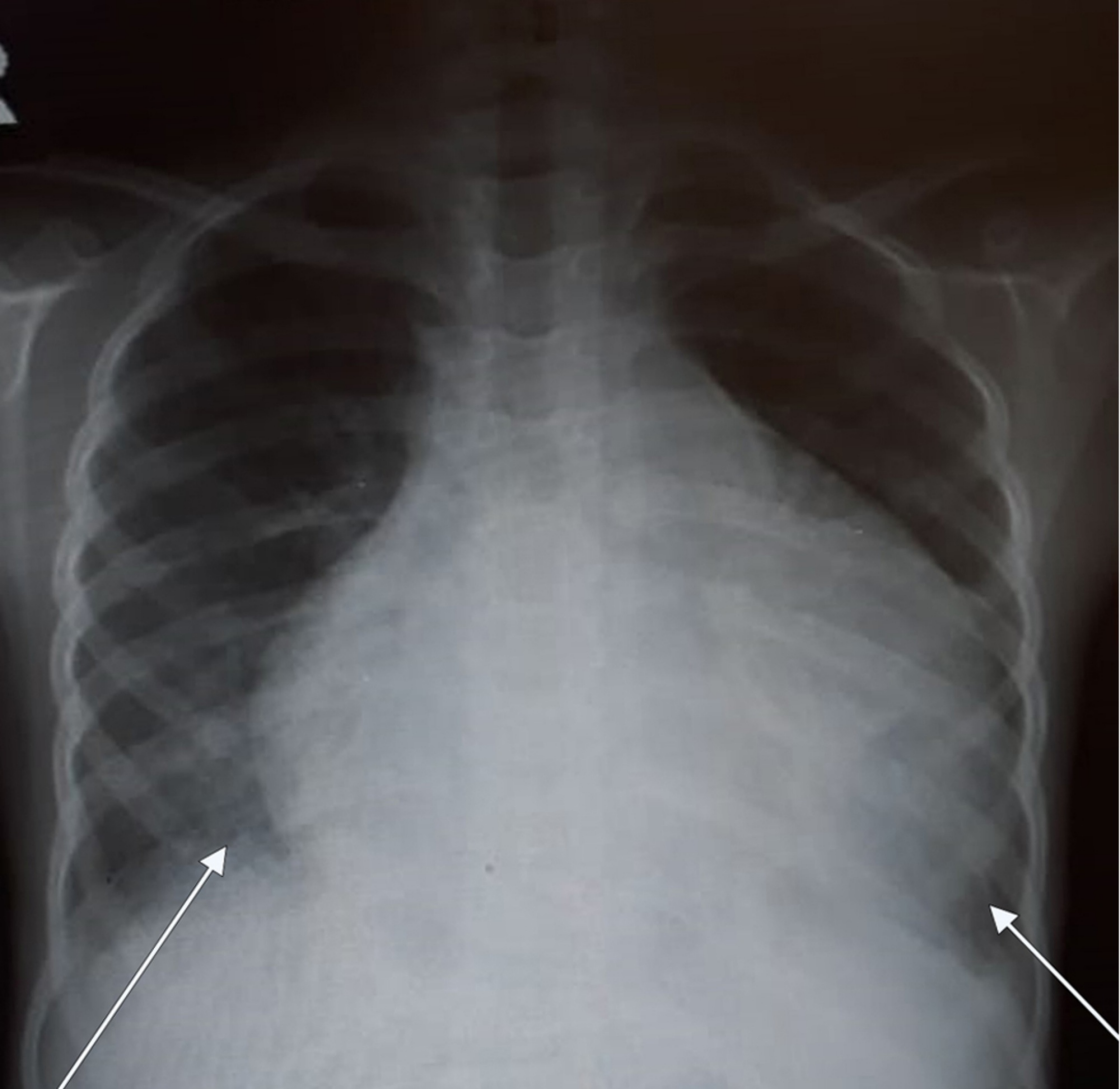
Chest radiograph showing enlarged cardiac silhouette

Chest radiograph showed an enlarged cardiac silhouette indicative of pericardial effusion (Figure 1). Echocardiography revealed a large collection of fluid around the posterior aspect of heart with atrial collapse strongly suggestive of cardiac tamponade.

Investigative analysis revealed that the patient was anemic and thrombocytopenic but with a normal leukocyte count. Erythrocyte sedimentation rate (ESR) was 92. Urine protein creatinine ratio was raised at 11.98 (reference, <0.3). Urine detailed report showed proteinuria and hematuria. Blood urea trend was recorded for six days which revealed an increasing pattern starting at 149 mg/dL on the first day to 168 mg/dL on the last day (reference range, 10 to 40 mg/dL). Creatinine levels showed a decreasing six-day pattern ranging from 1.49 mg/dL at first day to 1.03 mg/dL on the sixth day (reference range, 0.2 to 1.1 mg/dL). These lab values were suggestive of the autoimmune root of the disease and the renal dysfunction secondary to it (Table 1).

**Table 1:** Series of urea and creatinine levels

Date	26/8/2018	28/8/2018	29/8/2018	30/8/2018	2/9/2018	4/9/2018
Urea (reference range, 10–40 mg/dL)	149 mg/dL	160 mg/dL	171 mg/dL	175 mg/dL	191 mg/dL	168 mg/dL
Creatinine (reference range, 0.2–1.1 mg/dL)	1.49 mg/dL	1.68 mg/dL	1.22 mg/dL	1.18 mg/dL	1.23 mg/dL	1.03 mg/dL

Apart from ANA and anti-dsDNA positivity, the patient's serum came out positive for anti-Ro/SSA (anti-Sjogren's-syndrome-related antigen A, also called anti-Ro, or the combination anti-SSA/Ro or anti-Ro/SSA autoantibodies) and anti-La/SSB antibodies. Direct Coombs test was also positive. Serum complement levels were low with a C3 of 45 mg/dL (reference range, 101 to 186 mg/dL) and C4 of 8 mg/dL (reference range, 16 to 47 mg/dL). Thus, confirming the diagnosis of cSLE with cardiac tamponade.

As a management and definitive diagnostic tool, a fluoroscopy-guided pericardiocentesis was performed without any delay. A total of 430 ml of fluid was drained which was hemorrhagic; cytological analysis of the fluid was negative for malignancy with 60% neutrophils and 40% lymphocytes. Infectious workup was negative for acid-fast bacilli, any growth, or vegetation. Renal biopsy was performed to confirm the underlying cause of renal malfunction, as hypertension and proteinuria was noticed. Post-pericardiocentesis echocardiogram showed trace pericardial effusion and an ejection fraction of 35% with overall improvement.

The patient was managed for lupus carditis and nephritis and started on pulse therapy of intravenous methylprednisolone (500 to 1000 mg). This was followed by a low dose regime and treatment with mycophenolate mofetil starting on the seventh day which was aimed at reducing renal disease progression. The patient was continuously provided with organ support care such as positive pressure ventilation, hemodialysis, and invasive cardiovascular monitoring along with the instillation of intravenous fluid supplements. The patient improved significantly, was discharged from our hospital, and asked to follow up.

## Discussion

SLE is a heterogeneous autoimmune disease with diverse and complicated systemic manifestations [7]. There is a double incidence of SLE in the pediatric population of developing countries as compared to developed countries (16% - 23.5%) [8]. Despite a high occurrence index, there is a delay in the diagnosis of many cases, probably due to the variable clinical presentation of SLE [9]. SLE can involve any organ of the body; however, it is reported that childhood SLE has worse progression of the disease as compared to adults [1].

A retrospective study from Karachi, Pakistan reports that the most common presentation of SLE included fever (53%), malar rash (29%), photosensitivity (6%), arthropathy (38%), and renal involvement (33%) [10]. Pericarditis is the most common sign of lupus carditis; approximately 25% of patients with cardiac concurrence have pericarditis as a significant feature on echocardiogram [11]. In cSLE, the most commonly reported cardiac lesion is myocarditis. However, the incidence of cardiac lesions in cSLE is lower compared to adult SLE. Conversely, our patient presented with cardiac tamponade as an initial presentation which is a rare finding in the setting of lupus carditis and even rarer in childhood SLE [12]. Moreover, our patient does not have typical cardiac tamponade but sub-acute cardiac tamponade. SLE does present with both acute and sub-acute cardiac tamponade but the incidence of the latter is much lower.

A retrospective study of 409 patients revealed that only 5.9% of patients had SLE accompanied with cardiac tamponade [13]. A French study showed a picture of diverse and often severe initial manifestations seen in cSLE. It is recommended that SLE should be made a differential immediately in any febrile adolescent with unexplained organ involvement [14].

Pediatric patients with complement deficiencies can present with SLE-like manifestations but they usually present with rashes, glomerulonephritis, and rheumatic fever [15].

The clinical and immunological findings in our patient satisfied the SLICC classification criteria for SLE [1]. However, in addition, our patient presented with muffled heart sounds, tachycardia, and hypertension. Hypertension is common in cases of sub-acute tamponade and hypotension is common in cases of acute tamponade. Although tachycardia and decreased heart sounds are the two features that lack specificity and sensitivity for cardiac tamponade, these are nonetheless commonly reported. Classical signs of acute cardiac tamponade such as jugular venous distension, pulsus paradoxus, hypotension and dyspnea are not always seen in sub-acute tamponade due to a variety of reasons, such as assessment of jugular venous distention being observer-dependent, and pulsus paradoxus having low specificity and sensitivity for cardiac tamponade [16].

Thus we should rely on radiological investigations when cardiac tamponade is suspected. The most appropriate tool for diagnosing cardiac tamponade is echocardiography, which is preferable over cardiac catheterization due to its non-invasive approach. Echocardiography will show pericardial effusion and chamber collapse. Right ventricular collapse is more specific compared to right atrial collapse for cardiac tamponade; however, it is less sensitive [16]. In our case, atrial collapse is observed.

The most common management option for tamponade is pericardiocentesis. This procedure involves drainage of pericardial fluid with a 16 or 18 gauge needle inserted at an angle of 30 to 45 degrees to the skin near the left xiphoid margin. Pericardiocentesis works as a management option as well as a diagnostic means by which pericardial fluid can be analyzed, in order to reveal the underlying etiology of effusion [17].

Management of SLE includes combination therapy with steroids and immunosuppressants such as mycophenolate mofetil or azathioprine. This regime slows the disease progression and prevents organ damage mainly by inactivating the autoantibodies. A randomized trial of 340 patients which evaluated the efficacy of the aforementioned drugs, in combination with prednisolone in adolescents, revealed that mycophenolate mofetil was superior in preventing remission and kidney damage as a result of lupus nephritis [18].

The initial differential diagnosis for cSLE includes infections (sepsis, Epstein-Barr virus, parvovirus B19), malignancies, post-streptococcal glomerulonephritis, other rheumatologic conditions, and drug-induced lupus amongst others [1]. We kept SLE, vasculitis, and juvenile rheumatoid arthritis as a working diagnosis when the patient first presented to the hospital.

Tuberculosis remains the most common cause of pleural effusion in the pediatric population of South Asia [19]. The low serum levels of C4 complement are known to have a stronger association with cSLE as compared to SLE in adults. Low serum C4 levels and the young age of disease onset is found to be major predisposing factors towards pericarditis [20]. Serological, radiological, and clinical diagnostic measures should be used to identify the etiology of cardiac tamponade promptly in order to initiate appropriate treatment. Sub-acute tamponade does not always have the typical clinical presentation of cardiac tamponade, so keen observation and thorough investigation is important in these cases. Childhood SLE should be kept in differentials if a patient presents with prolonged fever, peripheral edema, rashes, signs of pericardial effusion, or any unexplained organ involvement especially in the presence of high ESR [14].

## Conclusions

Cardiac tamponade is a rare initial presentation in childhood SLE. Sub-acute cardiac tamponade is a less common form of tamponade which has more of an insidious onset. We report a case of childhood SLE with sub-acute cardiac tamponade as an initial presentation. Childhood SLE has a diverse pattern of clinical presentation, so it is crucial for physicians to identify cardiac tamponade as a probable indication of cSLE and proceed accordingly. Echocardiography and analysis of pericardial fluid are two important diagnostic measures whereas early recognition of cardiac tamponade can prevent morbidity and mortality as tamponade is known to affect pediatric population more severely than adults. The treatment options for cSLE include high dose steroid therapy with an immunosuppressant whereas pericardiocentesis is done for tamponade. Prognosis is good with prompt treatment.
